# Incubation system to assess gaseous emissions from environmental samples under controlled conditions

**DOI:** 10.1016/j.mex.2026.103852

**Published:** 2026-03-03

**Authors:** Lucilla Agostini, Hans-Martin Krause, Else K. Bünemann

**Affiliations:** a Research Institute of Organic Agriculture (FiBL), 5070 Frick, Switzerland; b Institute of Agricultural Sciences, ETH Zurich, 8092 Zurich, Switzerland

**Keywords:** Ammonia volatilization, Methane, Nitrous oxide, Carbon dioxide, Incubation

## Abstract

Gaseous emissions from agriculture need to be reduced to mitigate its environmental impact. In order to develop emission abatement strategies, systematic screening trials under controlled condition are an important basis for further evaluation in field trials. The Incubation System to Assess Gaseous Emissions (ISAGE) was designed to quantify emissions of greenhouse gases and ammonia from the same environmental sample (e.g. soil, fertilizers) with a versatile and compact setup. The ISAGE enables the user to:

Quantify methane, carbon dioxide, nitrous oxide and ammonia emissions from environmental samples

Switch between steady-state and non-steady state configurations without sample disturbance

Position up to 12 incubation units within the area of an A3 sheet

Comparable incubation setups have been employed in previous studies, but without providing sufficient technical specifications required for reproducibility nor validation data demonstrating their function. The present method provides detailed assembly instructions, describes a protocol to assess the performance of the incubation setup and reports data validating the functionality of the ISAGE. In fact, validation trials revealed that the measurement error for ammonia volatilization is dependent on incubation temperature, scrubbing time and headspace concentration. Thus, validity ranges and calibration equations are proposed to obtain bias-free NH_3_ volatilization estimates.

## Graphical abstract

**Figure:**
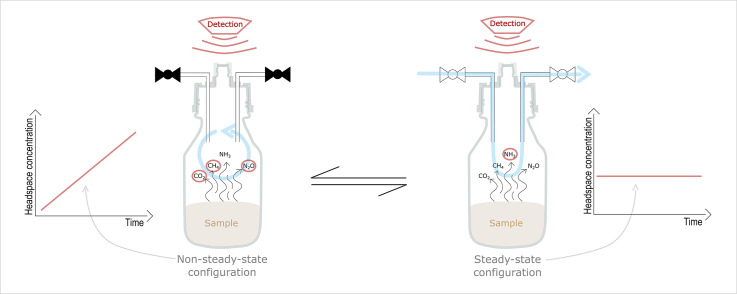

## Specifications table

**Table:** 

Subject area	Agricultural and Biological Sciences
More specific subject area	Gaseous emissions from environmental samples
Name of your method	Incubation System to Assess Gaseous Emissions (ISAGE)
Name and reference of original method	[1]. Impacts of feedlot floor condition, deposition frequency, and inhibitors on N_2_O and CH_4_ emissions from feedlot dung and urine patches. *Journal of the Air & Waste Management Association*, 68(7), 700–712. https://doi.org/10.1080/10962247.2018.1453392
Resource availability	A detailed list of component and files for 3D-printing are available at https://doi.org/10.5281/zenodo.15275632. Detailed protocols for sampling of emitted greenhouse gas and ammonia are available upon request.

## Background

Emissions of greenhouse gases (GHG) and ammonia (NH_3_) from the agricultural sector critically contribute to climate change and environmental pollution. An effective reduction of these emissions can only be achieved through a mechanistic understanding of their origin and implementation of effective mitigation strategies. When screening for management alternatives with lower climate impact, pilot trials under controlled conditions (i.e. in-vitro experiments) help to overcome limitations posed by field trials (i.e. in-situ experiments).

In-vitro experimental systems vary greatly in design, and most systems are tailored to a specific gas, quantification approach and sample type. For example, a common assessment approach for NH_3_ volatilization from environmental samples such as organically fertilized soil is the active scrubbing of NH_3_ emitted in a closed chamber into an acid solution followed by colorimetric quantification of ammonium (NH_4_^+^) [[2], [3], [4], [5], [6]]. Often, this configuration does not allow for sampling of other gases, such as GHGs, because of limited accessibility of the sample bottle headspace and limited flexibility of the setup to enable non-steady state conditions.

To assess methane (CH_4_), nitrous oxide (N_2_O) and NH_3_ emissions from dairy manure, Liao et al. [1] designed an experimental incubation system which allowed to alternate between a dynamic chamber arrangement for NH_3_ quantification and a static chamber arrangement for GHG quantification. However, their method description is not sufficient to replicate the proposed incubation set up. Furthermore, the uncertainties of NH_3_ quantification due to incomplete NH_3_ recovery in acid traps are not presented in this study. In fact, the strong propensity of NH_3_ for surface adsorption can lead to heavily biased estimates of NH_3_ volatilization. Thus, it is crucial to systematically quantify NH_3_ recovery deficits of a given experimental setup, but such data is rarely presented [3,7,6]. Furthermore, comparing NH_3_ volatilization estimates without considering NH_3_ recovery at detection implies the assumption of a linear relation between NH_3_ recovery at detection and NH_3_ volatilization. This may lead to biased conclusions, as NH_3_ surface sorption does not responding linearly to NH_3_ headspace concentration [8].

To address these challenges, the present method i) provides a detailed component list to replicate an incubation system to assess gaseous emissions from solid or liquid environmental samples under controlled conditions, ii) scrutinizes the assumption of linearity between NH_3_ recovery at detection and NH_3_ volatilization and iii) proposes a validation approach to debias NH_3_ volatilization estimates.

## Method details

### General design

The Incubation System to Assess Gaseous Emissions (ISAGE, ˈaɪs eɪdʒ) is designed to quantify emissions of multiple gases alternating between two measurement configurations: 1) the steady-state chamber design, where emitted gases are actively removed from the headspace by a steady gas flow, 2) the non-steady-state chamber design, where emitted gases accumulate in the headspace over time.

**Fig. 1:**
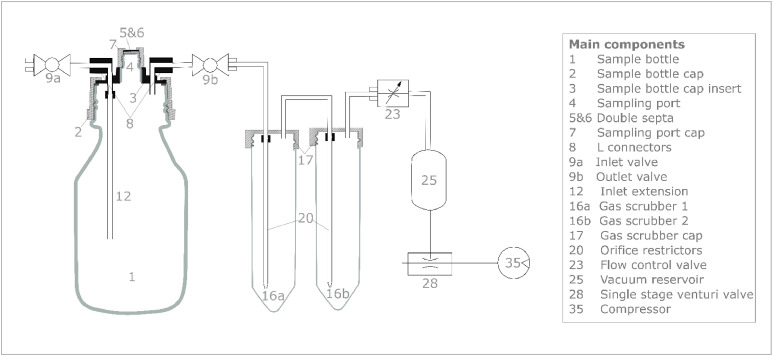
Schematic representation of the main components of a unit from the Incubation System to Assess Gaseous Emissions (ISAGE). Components are identified with an ID number and described in detail in SI 2. Components 25 (vacuum reservoir), 28 (single stage venturi valve) and 35 (compressor) are shared between multiple units.

The centrepiece of the incubation system is the sample bottle, which holds the sample throughout the incubation and assessment period (Fig. 1). The ISAGE is based on 250 mL screw-top bottles with GL 45 screw caps (DWK Life Sciences, D). The GL 45 screw cap is modified to feature an inlet, an outlet and a sampling port (detailed component list available at https://doi.org/10.5281/zenodo.15275632). In the non-steady-state configuration, the inlet and the outlet are closed by valves (Discofix® C, Braun, D) and the headspace is accessed through the sampling port by piercing a septum held in place by an exetainer head. In the steady-state configuration, the valves are opened to allow air circulation. An inlet extension directs inflowing air to the sample surface before being conveyed to the outlet. The air flow from the inlet to the outlet is generated by pulling a vacuum from the outlet with a single stage venturi valve (VN-05-N-T3-PQ2-VQ2-RQ2 Festo, D) powered by compressed air at ca. 40 psi. To minimize air flow fluctuations caused by back pressure inconsistency, orifice restrictors and a vacuum reservoir are included in the design. A flow control valve enables to regulate the air flow individually for each sample bottle. All ISAGE components are connected using PTFE tubing (4 mm ID) to minimize gas adhesion to the tubing surface [8].

Two gas scrubbers with 50 mL filling capacity each are inserted between the outlet of the sample bottle and the vacuum generator to avoid reintroduction of emitted gases to the incubation environment and prevent cross contamination between samples when measuring with the steady-state configuration. The solution in the gas scrubbers is adapted to the sampling purpose, e.g. with acid solution to trap NH_3_.

**Fig. 2:**
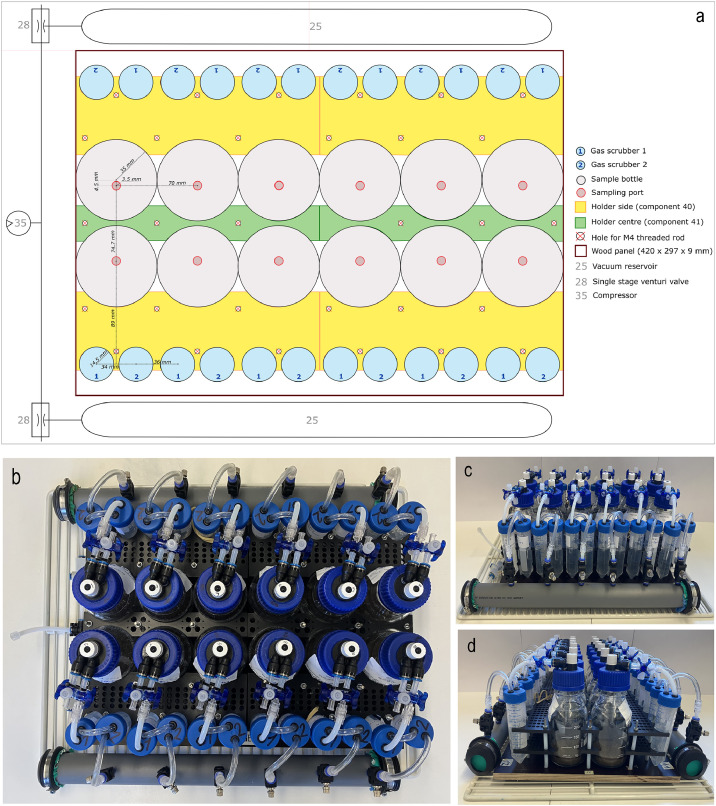
Schematic top view (a), photographic top view (b) and side views (c and d) of a 12-unit set of the Incubation System to Assess Gaseous Emissions (ISAGE). Sample bottles and gas scrubbers are held in place by two pairs of holders in the centre and four pairs of holders on the sides. Holder pairs are stacked with a distance of 2.5 cm between each other (for e. g. using PTFE tubing for spacing) and secured to a wooden panel using M4 threaded rod and nuts. Vacuum reservoirs and single stage venturi valves are shared between 6 units. The compressor is shared between all units.

For sample replication and treatment comparison, 24 ISAGE units are arranged in two sets of twelve units each. Each ISAGE set fits on an area of 440 mm x 317 mm, and the ISAGE units are held in place by custom 3D printed structures fixed on a 12 mm laminated board (Fig. 2). Each ISAGE set includes two vacuum reservoirs (2 L) and two single stage venturi valves shared between six ISAGE units. All single stage venturi valves are powered by the same compressed air source. To maintain constant temperature during incubation, ISAGE sets are located in an incubator (e.g. TS 606/3, WTW, D).

### Ammonia emissions

NH_3_ volatilization is quantified with the steady-state configuration. The headspace turnover rate is set by adjusting the output pressure of the compressor and the negative pressure applied to each sample bottle individually using the flow control valve. For example, a headspace turnover rate of 1 min^-1^ is achieved with a gas flow of 213 mL min^-1^ for a sample volume of 100 mL in a 250 mL screw-top bottle. To ensure comparable air flows across all ISAGE units during NH_3_ detection, the air flow is measured at the sample bottle inlet and adjusted with the flow control valve at the beginning of each scrubbing event.

Detection time and headspace turnover rate can be adjusted by the user to accommodate the experimental design and detection system. However, it is important to consider the water loss from the sample due to ventilation of the sample bottle. To avoid artificially altering the magnitude of NH_3_ volatilization, changes in sample water content should be kept below a chosen threshold (e. g. 5 % of initial water content). To achieve this, water loss is monitored gravimetrically and the original water content of samples is regularly restored with the addition of demineralized water.

The quantification of NH_3_ volatilization can occur either directly relying on a laser spectrometer or a photoacoustic sensor, or indirectly by scrubbing the headspace gas through gas scrubber 1 filled with an acid solution. Emitted NH_3_ is hereby trapped as ammonium (NH_4_^+^) which can be quantified performing the Berthelot reaction followed by spectrophotometry or by back titration. In both cases, detection has to occur before recirculating headspace gases back to the incubator through gas scrubber 2 which is filled with an acid solution to trap potential NH_3_ residues and prevent contamination of ambient air. So far, only the indirect quantification approach has been implemented for the ISAGE.

### Greenhouse gas emissions

GHG emissions can be quantified with the steady-state configuration relying on a laser spectrometer or a photoacoustic sensor with the same consideration on detection time and headspace turnover rates as described for NH_3_ measurements. Alternatively, GHG emissions can be assessed with the non-steady-state configuration, repeatedly sampling headspace gases over a given period and quantifying GHG concentration with a gas chromatograph. So far, only GHG quantification with the non-steady-state configuration has been implemented for the ISAGE.

To reliably quantify GHGs in the non-steady-state configuration, measurements are performed immediately after a headspace flushing period (steady-state configuration) of at least 1 h (e.g. for NH_3_ quantification). This ensures a low baseline of GHG concentrations. Repeated headspace sampling can occur manually or automatically. For manual sampling, samples are stored in evacuated exetainers before analysis and closure time is limited only by the sampling speed of the operator. Automated sampling (e.g. using a MultiPurposeSampler robotics smart series, Gerstel, D) can be combined with direct sample injection to the gas chromatograph, which however limits the minimum closure time based on the run time of the instrument. The gas chromatograph needs to be equipped with an electron capture detector (ECD) for N_2_O quantification and a flame ionization detector (FID) for CO_2_ and CH_4_ quantification.

To compensate for sample volume removed from the headspace in the non-steady-state configuration, the equivalent volume of an inert gas (e.g. He) should be added to the headspace before sample withdrawal for pressure equilibration. Failing to equilibrate headspace pressure can lead to overestimation of GHG emissions because of negative pressure.

## Method validation

### Ammonia emissions

Validation of NH_3_ volatilization measurements using the ISAGE was performed only for the indirect quantification approach. To assess the magnitude of NH_3_ losses in the ISAGE unit, calibration runs were performed with four sample NH_4__—_N concentrations (0 g N l^-1^, 0.2 g N l^-1^, 0.5 g N l^-1^, 1 g N l^-1^), three incubation temperatures (15 °C, 20 °C, 25 °C) and three scrubbing periods (1 h, 6 h, 12 h) in a full factorial design. Sample NH_4__—_N concentrations were simulated with a dilution series of (NH_4_)_2_SO_4 (aq)_. To initiate NH_3_ volatilization from 100 mL (NH_4_)_2_SO_4 (aq)_, 2 mL 1 mol l^-1^ NaOH were added immediately before starting scrubbing. In future experiments, however, it would be better to add NaOH to adjust the solution to a target pH (e.g., 9) and not in a fixed volume to account for the pH-lowering effect of elevated NH₄⁺ concentrations (> 0.5 g N l^-1^). Immediately after scrubbing termination, NH_3_ volatilization was stopped by adding 0.9 mL 1 mol l^-1^ H_2_SO_4_. At the beginning of each scrubbing event, headspace turnover rates of all ISAGE units were harmonized to 1 min^-1^ (213 mL min^-1^), measuring flow velocity at the sample bottle inlet (testo 405i, Testo, CH; with 4 mm tubing inner diameter, 213 mL min^-1^ are equivalent to 0.28 m s^-1^).

Gas scrubbers were filled with 30 mL 0.08 M sulfuric acid (H_2_SO_4_) each. The H_2_SO_4_ concentration was chosen according to the maximum amount of NH_3_ estimated to be emitted during the scrubbing period with an overhead of 100 % to account for the large uncertainty in estimation of NH_3_ volatilization. Initial and final NH_4__—_N concentrations in the sample bottles and the NH_4__—_N concentration in gas scrubber 1 were quantified performing the Berthelot reaction [9] followed by spectrophotometry (Genesys 150 UV–Vis, Thermo Fischer Scientific, USA).

Actual NH_3_ volatilization was calculated as the difference in NH_4__—_N concentration in the sample bottle at the start (NH_4__—_N_Start_) and the end (NH_4__—_N_End_) of the scrubbing period multiplied by the sample volume (100 mL) and normalized by emission area (0.003 m^-2^) and scrubbing time to obtain NH_3__—_N fluxes (Eq. (1)).

(1) $$Actual\;NH_{3}-N\;Flux=\;\frac{\left(NH_{4}-N_{Start}-\;NH_{4}-N_{End}\right)\times Sample\;Vol.}{Emission\;Area\;\times Scrubbing\;time}\;$$

Measured NH_3_ volatilization was equivalent to the NH_4__—_N concentration in gas scrubber 1 (NH_4__—_N_Gas Scrubber 1_) multiplied by the sample volume (30 mL) and normalized by emission area (0.003 m^-2^) and scrubbing time to obtain NH_3__—_N fluxes (Eq. (2)).

(2) $$Measured\;NH_{3}-N\;Flux=\;\frac{NH_{4}-N_{Gas\;Scrubber\;1}\times Sample\;Vol.}{Emission\;Area\;\times Scrubbing\;time}\;$$

Validity ranges were determined by testing the difference between means of actual and measured NH_3_ volatilization for each initial concentration, scrubbing time and incubation temperature with the Welch *t*-test (α = 0.05). For each combination of incubation temperature and scrubbing time, the minimum validity range was set to 0.0 g NH_3__—_N m^-2^ and the maximum to the highest mean measured NH_3_ volatilization, which was not statistically significant from the mean actual NH_3_ volatilization. Calibration equations were obtained applying linear regression between actual and measured NH_3_ volatilization within validity ranges.

### Validation results

**Fig. 3:**
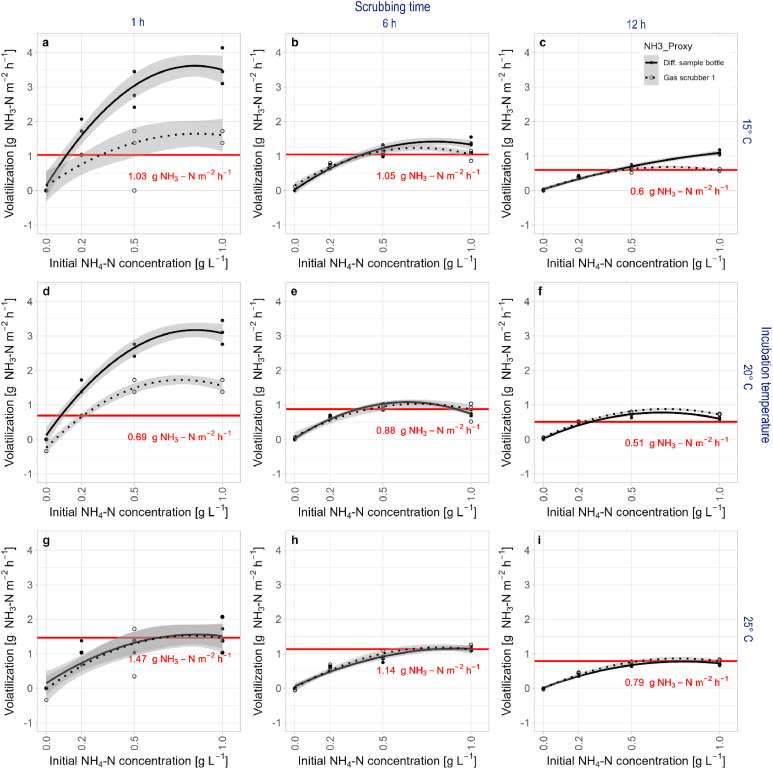
Ammonia nitrogen (NH_3—_N) fluxes from alkalinized ammonium sulfate solutions with four different initial ammonium nitrogen (NH_4—_N) concentrations measured for three different scrubbing times (1 h, 6 h, 12 h) and three different incubation temperatures (15 °C, 20 °C, 25 °C). Ammonia volatilization was quantified with two different estimators: NH_4__—_N content difference in the sample bottle before and after scrubbing (actual value) and NH_4__—_N content in gas scrubber 1 after scrubbing (measured value). Black lines represent quadratic regression with errors of the estimates as grey areas. Red lines and annotations indicate the mean NH_3__—_N emitted at the highest initial NH_4__—_N concentrations where the Welch *t*-test did not indicate significant differences between actual and measured values.

Actual NH_3_ volatilization increased with increasing initial NH_4__—_N concentration in the sample bottle following a hyperbolic saturation curve pattern (Fig. 3, solid markers). This effect derived from adding a fixed NaOH volume to differently concentrated (NH_4_)_2_SO_4_ solutions, resulting in different pH values and thus NH_3_ volatilization potentials. Longer scrubbing times resulted in significantly lower (*t*-test *p*-value < 0.05) actual NH_3_ volatilization, indicating a non-linear decrease of volatilization potential of alkalinized (NH_4_)_2_SO_4 (aq)_ over time. Actual NH_3_ volatilization was inversely proportional to incubation temperature, likely because of decreasing pH of (NH_4_)_2_SO_4 (aq)_ with increasing temperature. However, this trend was only statistically significant (*t*-test *p* = 0.01) when comparing volatilization after 1 h scrubbing time at 20 °C and 25 °C (Fig. 3d and Fig. 3*g*).

Measured NH_3_ volatilization responded to increasing initial NH_4__—_N concentration of samples with the same hyperbolic saturation curve pattern as actual NH_3_ volatilization. However, at lower incubation temperatures and scrubbing times, a disproportional underestimation of high actual NH_3_ volatilization was observed (Fig. 3, void markers). Higher incubation temperatures and scrubbing times reduced the underestimation of high actual NH_3_ volatilization, with a greater effect of incubation temperature than scrubbing time.

The variance of NH_3_ volatilization between samples with the same initial NH_4__—_N concentration (replicates) was not influenced by incubation temperature or scrubbing time (Levene's Test *p* > 0.05). The average coefficient of variance was 7 % for actual NH_3_ volatilization and 8 % for measured NH_3_ volatilization. No cross contamination was observed between samples, as indicated by measured NH_3_ volatilization from blanks (Table SI 1). The relative error of measured relative to actual NH_3_ volatilization across all initial NH_4__—_N concentrations was smallest with a scrubbing time of 6 h at 25 °C, but strongly varying between initial NH_4__—_N concentrations (Table SI 1). Validity ranges increased with incubation temperature because of lower relative errors at higher NH_3_ volatilization values, and they decreased with scrubbing time, as a result of decreasing volatilization potential of alkalinized (NH_4_)_2_SO_4 (aq)_ over time (Fig. 3, Table 1). Calibration equations derived from linear interpolation within validity ranges (Figure SI 1) show intercepts close to zero for all scrubbing times and incubation temperatures. Slopes differed most from one at 15 °C incubation temperature and 1 h scrubbing time. Increasing incubation temperature and scrubbing time brought slopes of calibration equations closer to one (Table 1). Coefficients of determination were above 0.93 except for scrubbing times 6 h at 20 °C and 1 h at 25 °C.

**Table 1:** Intercept, slope and coefficient of determination (R^2^) for correction of measured ammonia (NH_3__—_N) fluxes at three different incubation temperatures and scrubbing times within validity ranges. Validity ranges were determined by testing difference of means of actual (Diff. sample bottle) and measured (Gas scrubber 1) NH_3__—_N volatilization using the Welch *t*-test. Sample counts considered for linear interpolation (n) differ as a result of the defined validity ranges.

Incubation temperature [ °C]	Scrubbing time [h]	Validity range [measured g NH3-N m-2 h-1]	n	Intercept	Slope	R2
15	1	0.00 – 1.03	8	0.00	1.83	0.98
15	6	0.00 – 1.05	12	−0.17	1.29	0.93
15	12	0.00 – 0.60	12	0.01	1.08	0.94
20	1	0.00 - 0.69	8	0.46	1.65	0.94
20	6	0.00 – 0.88	12	0.02	0.95	0.87
20	12	0.00 – 0.51	8	0.04	0.98	0.99
25	1	0.00 – 1.47	12	0.27	0.78	0.73
25	6	0.00 – 1.14	16	0.03	0.93	0.96
25	12	0.00 – 0.79	16	0.02	0.88	0.98

Validation results showed how the ISAGE can be used to reliably measure NH_3_ volatilization under controlled conditions within the validity range for the chosen incubation temperature and scrubbing time and given the use of a calibration equation to debias measured NH_3_ volatilization within validity ranges (Table 1, Fig. 3).

### Greenhouse gas emissions

Measurement of GHGs using the non-steady-state configuration of the ISAGE requires appropriate closure times to avoid headspace saturation, homogenization of headspace before sample withdrawal, equilibration of negative headspace pressure resulting from sample withdrawal and suitable detection instrument accuracy and precision [10]. All these requirements are specific to the given environmental samples and experimental design. Thus, authors did not regard a general validation to be appropriate and recommend following GHG measurement guidelines proposed by the Global Research Alliance [11].

## Limitations

The ISAGE is suited for relative quantification of GHG and NH_3_ emissions (i.e. treatment vs control contrasts) rather than absolute quantification. Main reasons are artificial measurement conditions and systematic measurement errors.. In fact, NH_3_ volatilization is underestimated, particularly at low incubation temperatures and scrubbing times. Thus, defining validity ranges and correcting measured NH_3_ volatilization with a calibration equation determined for specific incubation conditions is crucial. Underestimation of measured NH_3_ volatilization could be mitigated by increasing the headspace turnover rate. However, this implies a trade-off between increasing NH_3_ recovery at detection and artificially increasing actual NH_3_ volatilization driven by the higher air flow across the sample surface.

Quantification of high GHG fluxes is limited by the relatively small and fixed headspace of the sample bottle. Thus, the selection of closure times and consequently of the sampling approach (manual vs automated) can be restricted by the necessity to avoid headspace saturation.

Although quantification of NH_3_ volatilization using gas scrubbers and of GHGs in the non-steady-state configuration provides the most resilient approach against data loss due to instrument failure (sample storage), time resolution of measurements is low and the need for human resources high. This limitation could be overcome by gas detection supported by laser spectrometers or photoacoustic sensors.

## Ethics statements

None

## CRediT author statement

**Lucilla Agostini**: Conceptualization, Methodology, Validation, Investigation, Writing original draft, Visualization **Hans-Martin Krause**: Conceptualization, Supervision, Review & Editing **Else K. Bünemann**: Funding acquisition, Conceptualization, Supervision, Review & Editing

## Related research article

None

## For a published article

None

## Declaration of competing interest

The authors declare that they have no known competing financial interests or personal relationships that could have appeared to influence the work reported in this paper.

## Data Availability

Data will be made available on request.

## Appendix Supplementary materials

Image, application 1
