# Supplementary material for: Incubation system to assess gaseous emissions from environmental samples under controlled conditions
Source: MethodsX. 2026 Mar 3;16:103852. doi: 10.1016/j.mex.2026.103852 (PMC12992078; doi:10.1016/j.mex.2026.103852)
Supplement: Supplementary file 1 [file mmc1.docx]

**Supplementary material *and/or* additional information [OPTIONAL]**


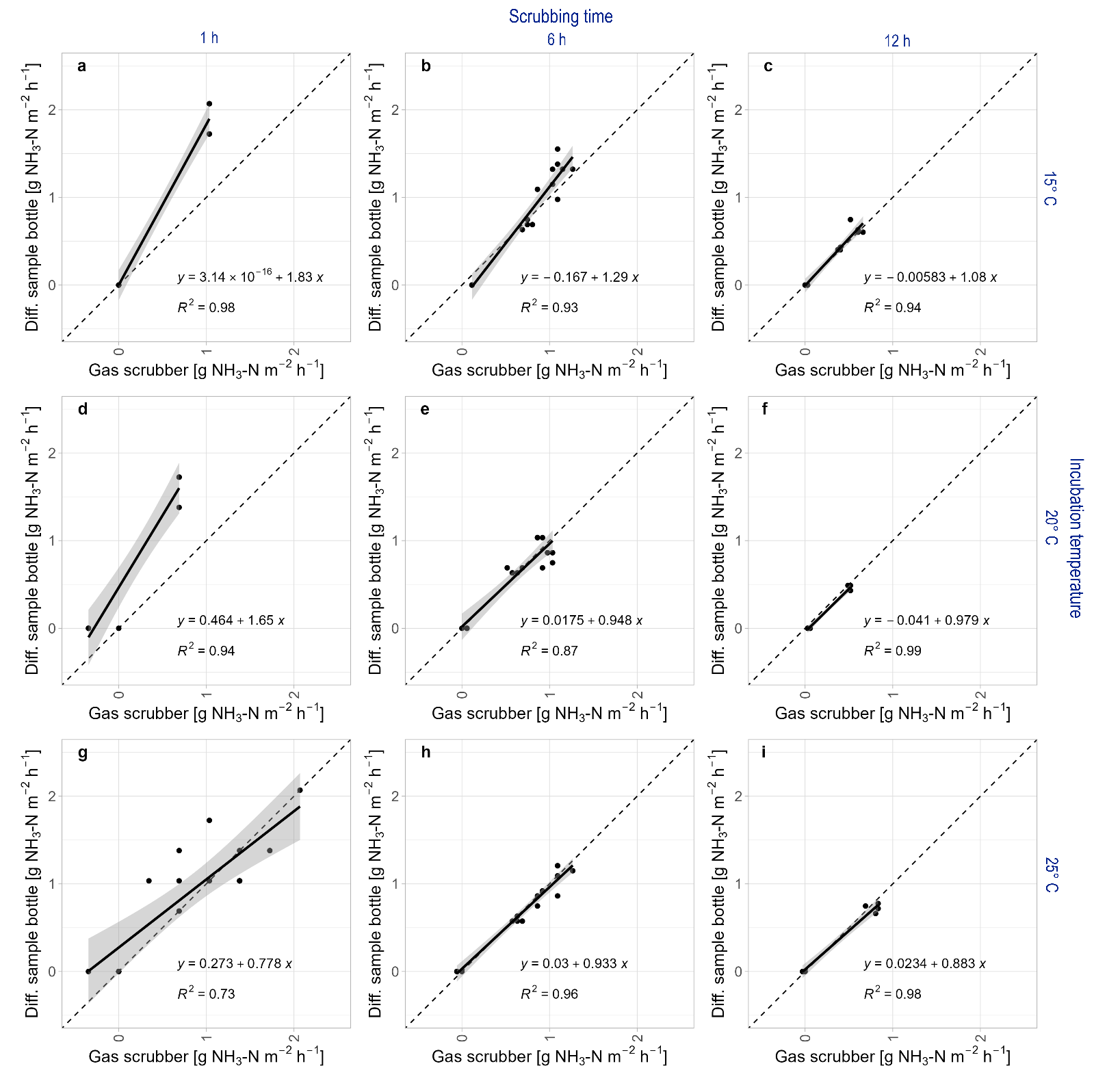


Figure SI 1: Volatilized ammonia nitrogen (NH_3_-N) from alkaline ammonium sulfate solutions with four different initial ammonium nitrogen (NH_4_-N) concentrations measured at three different incubation temperatures and scrubbing times. Ammonia volatilization was quantified with two different estimators: NH_4_-N content difference in the sample bottle before and after scrubbing (actual value) and NH_4_-N content in gas scrubber 1 after scrubbing (measured value). The relationship between actual and measured NH_3_ volatilization was modelled by linear regression (black regression line, grey standard error of the estimate). Only values within the validity range (Welch t-test) were considered. The dashed line represents the identity line.

Table SI 1: Mean ± standard deviation (n = 4) of actual ammonia (NH_3_) volatilization, measured NH_3_ volatilization and the relative error (relative to actual NH_3_ volatilization) measured at three different incubation temperatures and scrubbing times.

| Incubation temperature [°C] | Scrubbing time [h] | Actual NH_3_ volatilization [g NH_3_-N m^-2^ h^-1^] | Measured NH_3_ volatilization [g NH_3_-N m^-2^ h^-1^] | Relative Error [% of actual NH_3_ volat.] |
| --- | --- | --- | --- | --- |
| 15 | 1 | < 0.05 | < 0.05 | - |
| 15 | 1 | 1.9 ± 0.2 | 1.0 ± 0.0 | 45.0 ± 5.8 |
| 15 | 1 | 2.8 ± 0.4 | 1.2 ± 0.8 | 59.4 ± 27.7 |
| 15 | 1 | 3.5 ± 0.4 | 1.6 ± 0.2 | 52.8 ± 9.6 |
| 15 | 6 | < 0.05 | 0.1 ± 0.0 | - |
| 15 | 6 | 0.7 ± 0.0 | 0.7 ± 0.0 | 8.5 ± 6.8 |
| 15 | 6 | 1.2 ± 0.2 | 1.1 ± 0.1 | 12.0 ± 7.2 |
| 15 | 6 | 1.3 ± 0.2 | 1.0 ± 0.1 | 21.1 ± 6.8 |
| 15 | 12 | < 0.05 | < 0.05 | - |
| 15 | 12 | 0.4 ± 0.0 | 0.4 ± 0.0 | 5.2 ± 3.5 |
| 15 | 12 | 0.6 ± 0.1 | 0.6 ± 0.1 | 11.2 ± 13.6 |
| 15 | 12 | 1.1 ± 0.1 | 0.6 ± 0.0 | 46.3 ± 3.9 |
| 20 | 1 | < 0.05 | < 0.05 | - |
| 20 | 1 | 1.6 ± 0.2 | 0.7 ± 0.0 | 57.5 ± 5.0 |
| 20 | 1 | 2.5 ± 0.2 | 1.5 ± 0.2 | 41.5 ± 2.7 |
| 20 | 1 | 3.1 ± 0.3 | 1.6 ± 0.2 | 49.7 ± 7.3 |
| 20 | 6 | < 0.05 | < 0.05 | - |
| 20 | 6 | 0.6 ± 0.0 | 0.6 ± 0.1 | 4.5 ± 5.2 |
| 20 | 6 | 1.0 ± 0.1 | 0.9 ± 0.1 | 14.4 ± 2.7 |
| 20 | 6 | 0.7 ± 0.1 | 0.9 ± 0.2 | 29.2 ± 8.3 |
| 20 | 12 | < 0.05 | < 0.05 | - |
| 20 | 12 | 0.5 ± 0.0 | 0.5 ± 0.0 | 11.5 ± 10.1 |
| 20 | 12 | 0.7 ± 0.0 | 0.8 ± 0.0 | 13.7 ± 6.3 |
| 20 | 12 | 0.6 ± 0.0 | 0.7 ± 0.0 | 20.5 ± 5.6 |
| 25 | 1 | < 0.05 | < 0.05 | - |
| 25 | 1 | 1.0 ± 0.3 | 0.8 ± 0.2 | 20.8 ± 25.0 |
| 25 | 1 | 1.1 ± 0.2 | 1.2 ± 0.6 | 39.6 ± 18.5 |
| 25 | 1 | 1.6 ± 0.4 | 1.5 ± 0.4 | 18.3 ± 21.3 |
| 25 | 6 | < 0.05 | < 0.05 | - |
| 25 | 6 | 0.6 ± 0.0 | 0.6 ± 0.0 | 7.5 ± 9.6 |
| 25 | 6 | 0.8 ± 0.1 | 0.9 ± 0.1 | 10.5 ± 13 |
| 25 | 6 | 1.2 ± 0.1 | 1.1 ± 0.1 | 7.3 ± 4.8 |
| 25 | 12 | < 0.05 | < 0.05 | - |
| 25 | 12 | 0.4 ± 0.0 | 0.4 ± 0.0 | 17.9 ± 8.7 |
| 25 | 12 | 0.7 ± 0.0 | 0.7 ± 0.0 | 11.0 ± 4.3 |
| 25 | 12 | 0.7 ± 0.0 | 0.8 ± 0.1 | 13.2 ± 6.9 |
